# Safety and effectiveness of additional triamcinolone acetonide with endoscopic radial incision and cutting for benign stenosis of the lower gastrointestinal tract: A pilot study

**DOI:** 10.1002/deo2.70002

**Published:** 2024-09-03

**Authors:** Rintaro Moroi, Kotaro Nochioka, Satoshi Miyata, Hideya Iwaki, Hirofumi Chiba, Hiroshi Nagai, Yusuke Shimoyama, Takeo Naito, Hisashi Shiga, Masaki Tosa, Yoichi Kakuta, Shoichi Kayaba, Seiichi Takahashi, Yoshitaka Kinouchi, Atsushi Masamune

**Affiliations:** ^1^ Division of Gastroenterology Tohoku University Hospital Miyagi Japan; ^2^ Clinical Research Innovation and Education Center Tohoku University Hospital Miyagi Japan; ^3^ Teikyo University Graduate School of Public Health Tokyo Japan; ^4^ Division of Gastroenterology Iwate Prefectural Isawa Hospital Iwate Japan; ^5^ Division of Gastroenterology Iwaki City Medical Center Fukushima Japan

**Keywords:** endoscopic dilation, endoscopic radial incision, and cutting, inflammatory bowel disease, intestinal stricture, triamcinolone acetonide

## Abstract

**Objectives:**

Radial incision and cutting (RIC) is being investigated as an alternative endoscopic dilation method for lower intestinal tract stenosis, providing a high technical success rate and improving subjective symptoms. However, several patients develop re‐stenosis following RIC. In this pilot study, we aimed to evaluate the safety and efficacy of triamcinolone acetonide (TA) addition after RIC.

**Methods:**

RIC with TA was performed in 20 patients with lower gastrointestinal tract stenosis. We evaluated the rate of adverse events 2 months after RIC with TA. We investigated the short‐ and long‐term prognoses, as well as the improvement in subjective symptoms, using a visual analog scale.

**Results:**

The delayed bleeding rate after RIC was 23.8%. Endoscopic hemostasis was achieved in all patients with delayed bleeding. No perforations were observed. The cumulative re‐stenosis‐free, re‐intervention‐free, and surgery‐free rates 1 year after RIC were 52.9%, 63.7%, and 85.2%, respectively. Subjective symptoms, including abdominal pain, abdominal bloating, nausea, and dyschezia, significantly improved after RIC with TA.

**Conclusion:**

Although additional TA administration after RIC could be safe, additional TA may not be effective on luminal patency after dilation. Further investigation is warranted.

## INTRODUCTION

Benign stenosis of the lower gastrointestinal tract is a common complication that leads to bowel obstruction and may have a negative impact on quality of life. Various etiologies, including surgical anastomosis due to Crohn's disease (CD)^1^, ^2^ and colorectal tumor^3^ could develop such stenosis. Mucosal healing in inflammatory bowel disease (IBD)^4^ and other causes^5^, ^6^ can lead to intestinal strictures.

Endoscopic balloon dilation (EBD) is commonly used to treat benign stenosis.^7^, ^8^ Surgical treatments involving intestinal resection^9^ or strictureplasty^10^ have also been performed for EBD‐resistant lesions resistant to EBD. These treatment modalities have both advantages and disadvantages. We conducted two clinical trials to investigate the efficacy of a new endoscopic treatment called radial incision and cutting (RIC).^11^, ^12^ RIC has several advantages, such as a high technical success rate, and contributes to the resolution of subjective symptoms. However, several patients develop re‐stenosis following RIC. Our previous study reported that the cumulative re‐intervention‐free rate at one year after RIC was 55.7%.^12^ A countermeasure to prevent re‐stenosis is necessary.

Several studies have reported that triamcinolone acetonide (TA) may prevent re‐stenosis after esophageal circumferential endoscopic submucosal dissection (ESD).^13^, ^14^, ^15^, ^16^ However, no studies have reported a combination therapy of RIC and TA. Furthermore, TA has not yet been approved for the treatment of lower gastrointestinal strictures in Japan. Therefore, we conducted this exploratory study to investigate the safety and feasibility of TA after RIC.

In this study, we aimed to investigate the safety and feasibility of local TA administration to prevent re‐stenosis after RIC for lower gastrointestinal tract stenosis.

## METHODS

The protocol for this study has been published.^17^ Here, we provide an overview of the methodology used in this study.

### Study design and settings

This prospective, single‐arm, interventional, multicenter trial included three hospitals: Tohoku University Hospital, Iwaki City Medical Center, and Iwate Prefectural Isawa Hospital.

### Patients

Between June 2022 and September 2023, We enrolled 20 patients with lower intestinal strictures who underwent RIC between June 2022 and September 2023. The inclusion criteria were the same as in our previous report^11^, ^12^ and were as follows: (i) the presence of an intestinal stricture through which a colonoscope (approximately 10–12 mm in diameter) could not pass; (ii) the stricture was benign (not malignant); (iii) the length of the stricture was ≤ 2 cm (estimated under fluoroscopy); and (iv) no abscess or intestinal fistula near the stricture. Intestinal strictures were classified into two types according to the cause of formation: (i) primary stricture due to mucosal healing after intestinal inflammation and (ii) secondary stricture (anastomotic stricture after intestinal resection due to CD or colorectal tumor). Patients with both stricture types were included in the study.

We set the sample size at 20 cases for this study. The rationale for setting the sample size at 20 cases includes the following points: (i) previous reports of TA in the esophageal field have involved approximately 20 cases^13^, ^15^; (ii) our previous study^11^ included 20 cases; (iii) the study was designed as a preliminary investigation of safety.

### Procedures and devices for RIC and TA administration

**FIGURE 1:**
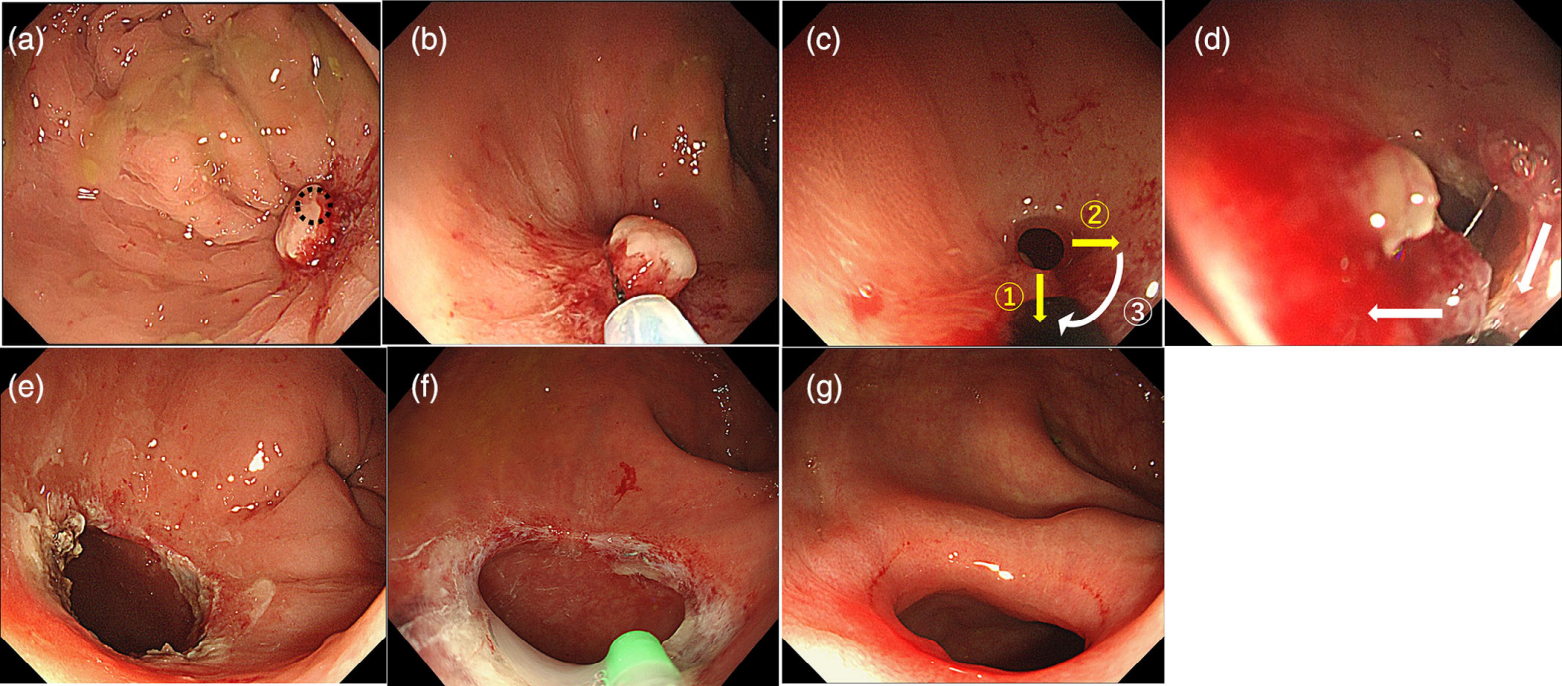
Procedure of radial incision and cutting (RIC). A male in his 50s after low anterior resection for rectal cancer. He was referred to our hospital due to the difficulties of endoscopic balloon dilation. (a) Anastomotic stenosis with granulomatous protruding lesion. Anastomotic stenosis (black circle) is invisible due to protruding lesions. (b) resection of protruding lesion before RIC via cold snare polypectomy technique. (c) Scheme of RIC. First and second radial incisions (yellow arrow) and subsequent horizontal cuts (white arrow) were performed. (d) Horizontal cut along with white allows. (e) Completion of RIC. (f) Administration of triamcinolone acetonide using a spray tube 1 week after RIC. (g) One year after RIC. A colonoscopy could pass through the dilation site.

A representative case of RIC and TA administration is shown in Figure 1. The RIC procedure was performed as described in our previous reports^11^, ^12^ using an electrosurgical endo‐knife with a small insulated tip (ITknife Nano; Olympus) for ESD. The primary scope was PCF‐H290TI (Olympus Medical Science). The settings of the electrosurgical unit (VIO300D; ERBE Electromedicine GmbH) were the same as those used for ESD of colonic tumors. (ENDO CUT I effect: 3, duration: 2, cutting duration: 2, SWIFT COAG effect: 3, 45 W). An endoscopist with > 50 colorectal ESD cases was eligible for RIC.

TA was locally administered at a dose of 50 mg per session with a spray tube two days after RIC, and 1 week after RIC. An injection needle was not used for administrating TA in this study. The composition of the TA dilution was uniform in all cases.

### Analysis and statistics

The primary endpoint was the safety of TA administration 2 months after RIC for lower gastrointestinal strictures. The safety of TA was evaluated by measuring the frequency of adverse events of special interest (AESI) during the study period. AESI includes RIC‐related complications such as delayed bleeding and perforation requiring endoscopic hemostasis or surgery. We defined delayed bleeding as (i) hematochezia after RIC that required endoscopic hemostasis, or (ii) a decrease in hemoglobin of 2.0 mg/dl or more. We set this primary endpoint because TA has not yet been approved for the treatment of lower gastrointestinal tract lesions in Japan. Furthermore, this was an exploratory study of TA administration after RIC.

The secondary endpoints were as follows: (i) scope passage rate at the dilation site 2 months after RIC; (ii) long‐term prognosis after RIC including the cumulative re‐stenosis free rate, re‐intervention free rate (defined as re‐RIC or EBD or surgery for symptomatic re‐stenosis), and surgery‐free rate analyzed using the Kaplan‐Meier method; (iii) the scope passage rate at 2 months after RIC; (iv) technical success rate of RIC (defined as the scope passage immediately after RIC) and procedure time; and (v) improvement in subjective symptoms (abdominal pain, nausea, abdominal bloating, and dyschezia) using a visual analog scale (VAS). The VAS is one of the scales used to evaluate subjective symptoms and is a 100 mm line.^18^ The patients marked the anchor on the line indicating the strength of their symptoms. The score was evaluated by measuring the distance from the start to the anchor and comparing it before and 4–8 weeks after RIC.

The timing of the scheduled endoscopy was 2 months after RIC. Additional endoscopies apart from scheduled ones were conducted based on the subjective symptoms including abdominal pain or participants’ requests. Another timing of endoscopy was 1 year after RIC.

Descriptive statistics were calculated for the primary and secondary outcomes. Continuous variables are presented as the mean ± standard deviation (SD). Categorical variables are presented as numbers and percentages. Improvement in subjective symptoms was evaluated using Welch's t‐test. The incidence rates of long‐term prognosis after RIC were estimated using Kaplan‐Meier curves. Statistical significance was defined as a two‐sided *p*‐value < 0.05. All analyses were performed using R version 4.3.3.^19^

### Ethics

The study protocol was approved by the Tohoku Certified Review Board of Tohoku University (Ministry of Health, Labor and Welfare Certified Clinical Research Review Board, Tohoku University; 2022‐0219). Written informed consent was obtained from all the patients. The current protocol was registered in the Japan Registry of Clinical Trials (registration no: jRCTs021220004; URL: https://jrct.niph.go.jp/re/reports/detail/23893).

## RESULTS

### Patients’ backgrounds

We conducted RIC for 20 lesions in 20 participants and performed 21 sessions. Patient backgrounds are summarized in Table 1. Sixteen patients had secondary strictures, and the remaining four had primary strictures. The etiology of stenosis varies, with the most frequent being surgery for CD. A history of EBD and stricture ulceration were observed in 10 and six patients, respectively. Nine participants received biologics for IBD treatment. We did not estimate the diameter of the stricture. Of six participants with ulceration at the stenosis site, four had CD (Infliximab: 1, Adalimumab: 1, Vedolizumab: 1, and non‐biologics: 1), one had ulcerative colitis (non‐biologics), and one had postoperative anastomotic stricture rectal cancer following rectal cancer surgery. On the contrary, among 14 participants with non‐ulceration at the stenosis site, eight had CD (Adalimumab: 3, Infliximab: 1, Vedolizumab: 1, and Non‐biologics: 3), and two had ulcerative colitis (Infliximab:1 and non‐biologics: 1).

**TABLE 1:** Backgrounds of the study population.

	*n* = 20
Sex (male:female)	15:5
Mean age (SD)	53.4 (15.3) years
Stenosis site, RIC sessions	20 lesions, 21 sessions
Primary:secondary	4:16
Etiologies of stenosis	Mucosal healing of UC: 2
	Mucosal healing of CD: 2
	Surgery due to CD: 10
	Surgery due to UC: 1
	Surgery due to colorectal tumor: 5
Location of stenosis	Ileum: 2
	Ileo‐colonic anastomosis: 10
	Colon: 7
	Ileal pouch: 1
Previous history of EBD	10
Ulceraton on the stenosis	6
Administration of medication for IBD	Infliximab: 3
	Adalimumab: 4
	Vedolizumab: 2
	Azathioprine: 2
	Steroid: 0

Abbreviations: EBD, endoscopic balloon dilation; CD, Crohn's disease; IBD, inflammatory bowel disease; RIC, radial incision and cutting; SD, standard deviation; UC, ulcerative colitis.

### Primary endpoint: Safety profile of RIC with TA administration

Table 2 summarizes the adverse events during the 2 months after RIC with TA. Of the 20 participants with 21 sessions of RIC, five sessions of RIC in four participants developed delayed bleeding (23.8%). All cases with delayed bleeding met definition (i) (requiring endoscopic hemostasis). Endoscopic hemostasis using an electrosurgical coagulation device was successfully performed in all patients with delayed bleeding. Delayed bleeding was observed in all cases of ileocolonic anastomosis due to CD. Perforations were not observed. One patient developed worsening CD, which led to re‐stenosis and subsequent surgical intestinal resection.

**TABLE 2:** Adverse events within the 2 months after radial incision and cutting with additional triamcinolone acetonide administration.

Adverse event	RIC with TA
Delayed bleeding	23.8% (5/21)
Worsening of CD	8.3% (1/12)
Perforation	0% (0/20)

Abbreviations: CD, Crohn's disease; RIC, radial incision and cutting; TA, triamcinolone acetonide.

### Long‐term prognosis of RIC with TA administration

**FIGURE 2:**
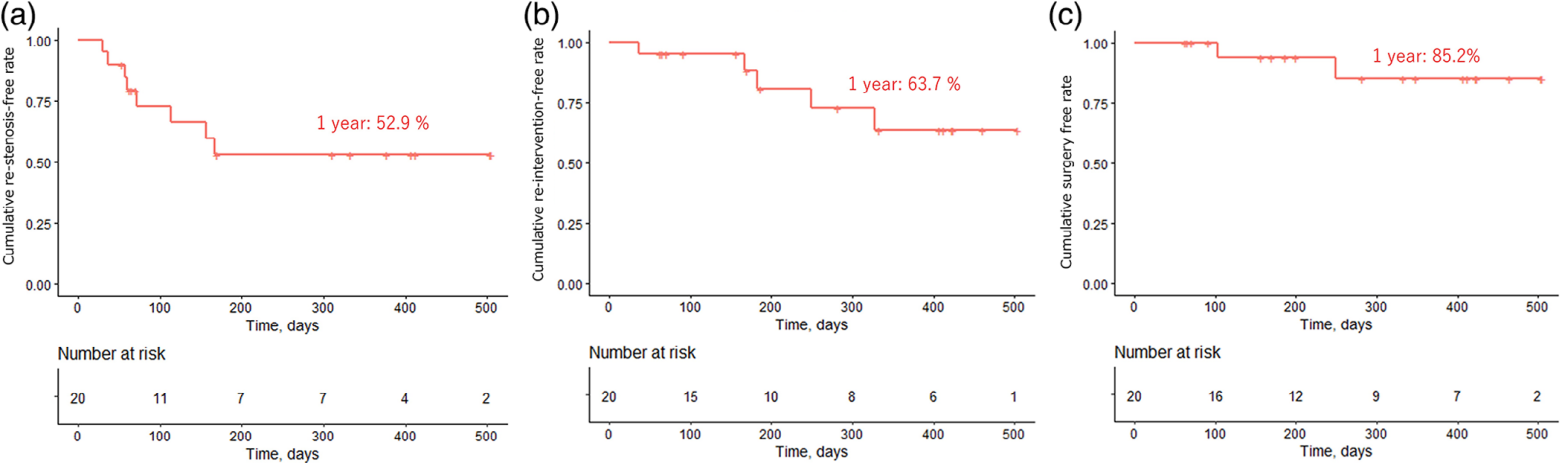
Long‐term prognosis of radial incision (RIC) and cutting with triamcinolone acetonide. (a) The cumulative re‐stenosis‐free rate at 1 year after RIC was 52.9%. (b) The cumulative re‐intervention‐free rate 1 year after RIC was 63.7%. (c) The cumulative surgery‐free rate at 1 year after RIC was 85.2%.

The long‐term outcomes of RIC with TA are shown in Figure 2. The cumulative re‐stenosis‐free, re‐intervention‐free, and surgery‐free rates at 1 year after RIC were 52.9%, 63.7%, and 85.2%, respectively. The cumulative re‐stenosis‐free and re‐intervention‐free rates at 6 months after RIC were 52.9% and 83.2%, respectively. Two cases undertook balloon dilation due to re‐stenosis within 6 months after RIC. One participant underwent re‐RIC 11 months after the first RIC, and two participants underwent surgery 1 and 8 months after RIC (Figure 2).

### Short‐term results of RIC

The short‐term results are presented in Table 3. The technical success rate of RIC was 100% (21/21). The mean procedure time for RIC was 15.1 min. The mean length of hospitalization after RIC was 12.1 days. All patients completed the RIC procedure and TA administration. The scope passage rate 2 months after RIC was 70%.

**TABLE 3:** Short‐term results of radial incision and cutting with additional triamcinolone acetonide administration.

	20 participants, 20 lesions, and 21 sessions of RIC
Technical success rate of RIC	100% (21/21)
Mean procedure time of RIC (SD)	15. 1 min (SD 9.9)
Hospital stay after RIC (SD)	12.1 days (SD 7.7)
The rate of scope passage 2 months after RIC	70% (14/20)

Abbreviations: RIC, radial incision and cutting; SD, standard deviation; TA, triamcinolone acetonide.

### Improvement of subjective symptoms after RIC

**FIGURE 3:**
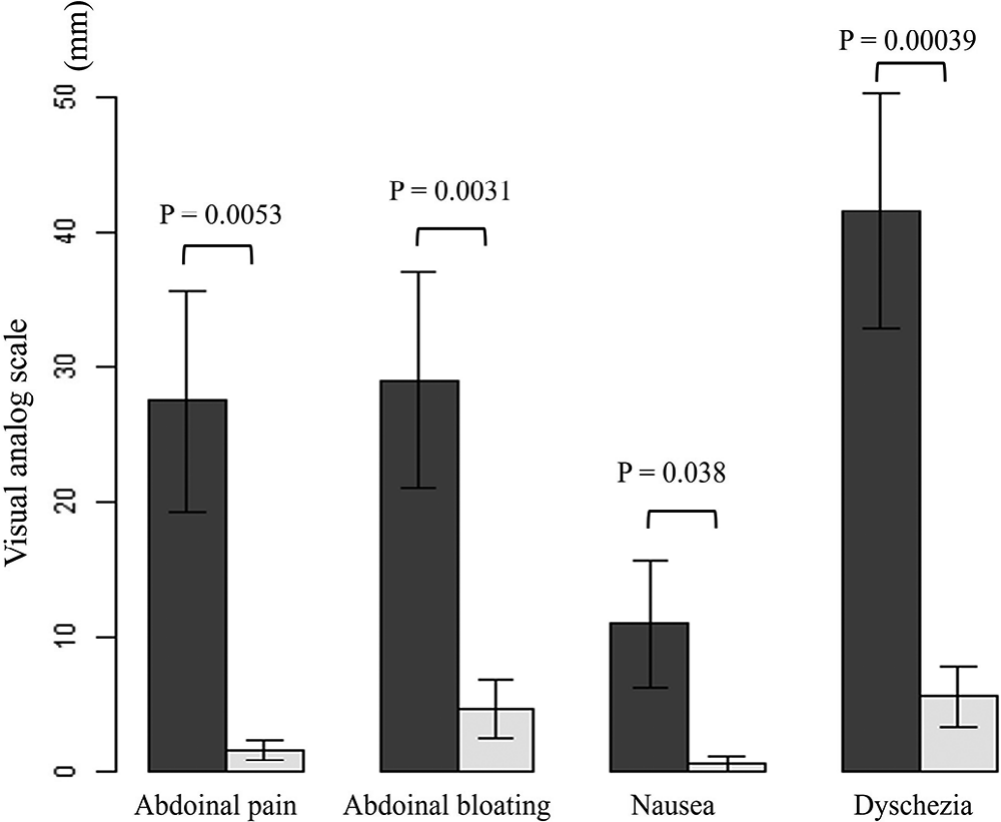
Comparisons of subjective symptoms before and 4–8 weeks after radial incision and cutting. Each symptom improved after radial incision and cutting.

The improvements in subjective symptoms are summarized in Figure 3. Each symptom including abdominal pain (27.5 mm vs. 1.6 mm, *p* = 0.0053), abdominal bloating (29 mm vs. 4.7 mm, *p* = 0.0031), nausea (11 mm vs. 0.61 mm, *p* = 0.038), and dyschezia (41.6 mm vs. 5.6 mm, *p* = 0.00039) was improved after RIC.

## DISCUSSION

In this study, we investigated the safety and efficacy of RIC combined with TA. The main adverse event associated with RIC combined with TA is delayed bleeding. Perforations were not observed. The cumulative re‐stenosis‐free, re‐intervention‐free, and surgery‐free rates at 1 year after RIC were 52.9%, 63.7%, and 85.2%, respectively. The technical success rate of RIC was 100%. RIC improved subjective symptoms.

The safety of additional TA administration after RIC for lower gastrointestinal strictures may not be problematic. Although statistical analysis was not performed, the rate of delayed bleeding was comparable to that reported in our previous study that investigated RIC without TA. Our previous studies^11^, ^12^ reported a delayed bleeding rate of approximately 20%, which was similar to the present study. Moreover, the delayed perforation rate in this study was 0%. An animal study reported that TA injection into the muscle layer could lead to perforation.^20^ However, other human studies have reported that local TA administration after esophageal ESD does not develop delayed perforation.^13^, ^14^, ^15^, ^16^ Our results, along with those of previous studies, indicate that additional TA administration after RIC may not negatively affect the short‐term clinical course. However, the small number of participants and the differing backgrounds and etiologies of stricture formation in each study preclude a direct comparison in the safety of TA. Therefore, further investigations with larger cohorts are necessary.

Additional TA administration may not have a positive effect on patency duration after RIC. Regarding the long‐term outcomes, the cumulative re‐intervention‐free rate at 1 year after RIC in this study was 63.7%, whereas that of RIC without TA in our previous study was 55.7%.^12^ The present study and our previous research demonstrated similar long‐term outcomes (Figure S1). This result indicates that additional TA may not lengthen the patency duration after RIC. However, the appropriate dose and method of TA administration remain unclear. First, the number of patients included in this study was relatively small. Furthermore, several studies have reported that TA administration after esophageal ESD prevented re‐stenosis.^13^, ^14^, ^15^, ^16^ Second, we did not use injection needles to administrate TA because we prioritized safety in this pilot study and avoided risks of perforation due to TA injection into the muscle layer.^20^ However, a short injection needle may be safe to administrate TA locally and is expected to be more effective in preventing re‐stenosis than a spray tube. As with the comparison of adverse events, we did not directly compare the effects of TA between this study and previous studies due to the small sample size and different backgrounds. Further investigations are required to determine the efficacy of TA for patency after RIC in the lower gastrointestinal tract. Other approaches should also be considered to prevent re‐stenosis after endoscopic dilation.

The short‐term outcomes of RIC are preferable, except for delayed bleeding. The technical success rate of RIC in the present study was 100%. Our previous studies on RIC^11^, ^12^ and similar studies on endoscopic dilation using an electric knife^21^, ^22^, ^23^ reported technical success rates of approximately 100%. This incision method could be an alternative for dilating intestinal strictures. Furthermore, combination therapy with RIC and EBD could also be considered as an alternative method for intestinal stricture. Dilation is typically performed using RIC or EBD alone. A dilation strategy that combines several incisions for the fibrotic part of the stricture and subsequent balloon dilation could be considered because it would be technically easy, even in cases where it is difficult to perform a circumferential incision. Such an approach may also contribute to lengthening the patency duration.

The impact of concomitant biologics administration for IBD on a restenosis‐free period following RIC was unclear. Although we performed RIC for strictures with ulceration, the number of participants with both ulceration at the stenosis site and biologics was small. Therefore, it may be difficult to discuss the impact of biologics based on the result of this study. Anti‐tumor necrosis factor‐α theoretically has a risk of stricture formation.^24^ However, several studies reported a positive impact of biologics clinical course following EBD^25^ and the healing process of small bowel ulcers.^26^ We believe that further investigations with more participants are necessary to evaluate the impact of biologics on the restenosis‐free period following RIC.

Our results showed that the rate of delayed bleeding after RIC was relatively high. In the present study, the rate of delayed bleeding was approximately 20%. TA did not increase delayed bleeding, and endoscopic hemostasis was achieved in all cases. However, a high rate of delayed bleeding may be problematic. All cases of delayed bleeding involved ileocolonic anastomoses associated with CD. Our previous study demonstrated a similar tendency in the etiology of stenosis formation.^11^, ^12^ Other types of stenosis, such as colonic anastomosis related to cancer surgery and ileum‐ileum anastomosis related to CD, did not lead to delayed bleeding. Delayed bleeding was observed only in patients with CD‐related ileocolonic anastomoses. Further investigation is required to reduce the risk of delayed bleeding.

This study had several limitations. First, selection bias in this study cannot be denied. This study included a small number of participants whose background, previous history, and treatment for IBD are heterogeneous and varied. This variation and small number of participants contributed to selection bias which may overestimate the safety of TA and underestimate its efficacy in preventing re‐stenosis. Further investigations with larger cohorts and stratified analysis according to the participants’ backgrounds are necessary to eliminate selection bias. Second, there is room for consideration when measuring TA administration. In this study, we administered TA using a spray tube, based on previous research.^27^ One study using an animal model reported the risk of perforation due to TA injection.^20^ Safety was prioritized because this was a pilot study. Third, RIC is not commonly used to treat lower gastrointestinal stenosis in Japan. Currently, RIC should be performed by an experienced endoscopist because this RIC technique is more difficult than EBD. However, RIC has the potential to dilate stenosis, even in cases of EBD‐resistant stenosis. The feasibility and safety of RIC should be validated by additional endoscopists and institutions from now on.

In conclusion, although additional TA administration after RIC could be safe, additional TA may not be effective on luminal patency after dilation. Therefore, further investigation is warranted.

## CONFLICT OF INTEREST STATEMENT

The authors declare no conflict of interest.

## Supporting information

FIGURE S1 Comparison of the cumulative re‐intervention‐free rate after RIC with and without TA.
